# Visualization of Actin Polymerization in Invasive Structures of Macrophages and Carcinoma Cells Using Photoconvertible β-Actin – Dendra2 Fusion Proteins

**DOI:** 10.1371/journal.pone.0016485

**Published:** 2011-02-14

**Authors:** Athanassios Dovas, Bojana Gligorijevic, Xiaoming Chen, David Entenberg, John Condeelis, Dianne Cox

**Affiliations:** 1 Anatomy and Structural Biology, Albert Einstein College of Medicine, Bronx, New York, United States of America; 2 Gruss-Lipper Biophotonics Center, Albert Einstein College of Medicine, Bronx, New York, United States of America; 3 Developmental and Molecular Biology, Albert Einstein College of Medicine, Bronx, New York, United States of America; The Beatson Institute for Cancer Research, United Kingdom

## Abstract

Actin polymerization controls a range of cellular processes, from intracellular trafficking to cell motility and invasion. Generation and elongation of free barbed ends defines the regions of actively polymerizing actin in cells and, consequently, is of importance in the understanding of the mechanisms through which actin dynamics are regulated. Herein we present a method that does not involve cell permeabilization and provides direct visualization of growing barbed ends using photoswitchable β-actin - Dendra2 constructs expressed in murine macrophage and rat mammary adenocarcinoma cell lines. The method exploits the ability of photoconverted (red) G-actin species to become incorporated into pre-existing (green) actin filaments, visualized in two distinct wavelengths using TIRF microscopy. In growing actin filaments, photoconverted (red) monomers are added to the barbed end while only green monomers are recycled from the pointed end. We demonstrate that incorporation of actin into intact podosomes of macrophages occurs constitutively and is amenable to inhibition by cytochalasin D indicating barbed end incorporation. Additionally, actin polymerization does not occur in quiescent invadopodial precursors of carcinoma cells suggesting that the filaments are capped and following epidermal growth factor stimulation actin incorporation occurs in a single but extended peak. Finally, we show that Dendra2 fused to either the N- or the C-terminus of β-actin profoundly affects its localization and incorporation in distinct F-actin structures in carcinoma cells, thus influencing the ability of monomers to be photoconverted. These data support the use of photoswitchable actin-Dendra2 constructs as powerful tools in the visualization of free barbed ends in living cells.

## Introduction

Actin polymerization is a dynamic and highly controlled process that regulates a range of cellular functions such as organelle transport, intracellular pathogen spread and cell motility. Actin filaments are polar, with polymerization occurring preferentially at the “plus” or “barbed” end. Barbed ends are generated by three distinct mechanisms: uncapping of pre-existing barbed ends (e.g. through phosphoinositide-mediated release of gelsolin [1]), de novo nucleation mediated by the Arp2/3 complex [2], formins [3] or Spire [4], and severing of filaments by ADF/cofilin proteins, the later mechanism creating barbed ends as well as a free pool of G actin available for re-polymerization [5].

Several methods for the visualization and quantitative analysis of actin polymerization in cells have been developed, but few provide an accurate measure of growth at free barbed ends. Time resolved permeabilization using detergents, followed by addition of labeled actin monomers and fixation of cells is one such method [6]. However, this method cannot be performed in living cells over time, and permeabilization may create artifacts, such as extraction of proteins important for polymerization, and removal or delocalization of filaments. Furthermore, the permeabilization may not be homogeneous when cells with a high membrane content and volume, such as macrophages, are analyzed (A.D. and D.C., unpublished data). Methods to monitor active transport of actin to the leading edge of live cells using fluorescent proteins fused to β-actin have been described, such as the use of photoactivatable Dronpa-actin [7] and FLAP (fluorescence localization after photobleaching) [8] but these methods may not directly measure barbed ends incorporation. The first method does not provide a reference structure as it requires bleaching of the entire fluorescent actin pool prior to photoactivation, while the second utilizes fusion of actin to two distinct fluorophores that are equally distributed throughout the cell at all times, making analysis difficult. Furthermore, our previously described method using EGFP-β-actin [9], although reliable and accurate, can only identify barbed ends during a burst of actin polymerization, i.e., when addition of monomers at the barbed end (which induces an increase in fluorescence) is much faster than the depolymerization (which induces a decrease in fluorescence) at the pointed end of the filament. Therefore, it does not extend the measurement of β-actin incorporation into barbed ends when the relative contributions of polymerization and depolymerization are comparable, such as in a treadmilling filament.

We believe that the use of photoconvertible β-actin – Dendra2 fusion proteins can be used to circumvent these problems. Namely, after the photoconversion of a small soluble population of actin monomers from green to red, red monomers will be added to the barbed end of the polymer, while unconverted monomers removed from the pointed end of the filament will only be green. As a result, fluorescence in the green channel will fluctuate both with polymerization and depolymerization of filaments whereas the fluorescence in the red channel will only detect polymerization. The green fluorescence provides the added advantage that it allows the visualization of pre-existing reference filaments onto which polymerization occurs.

We decided to test this approach in actin-rich structures found in macrophages and invasive carcinoma cells, namely podosomes and invadopodia respectively, which are linked to the ability of these cells to invade tissues during inflammation or cancer metastasis. Control of actin polymerization in these structures has therefore been the subject of intense study in recent years (reviewed in [10], [11]).

## Materials and Methods

### Constructs

The Dendra2 cDNA was amplified from the pDendra2-N vector using the following primers:

Forward: TTTAAA*AAGCTT*CCACCATGAACACCCCGGG (*HindIII*)

Reverse: TTTAAA*GGATCC*CCACACCTGGCTGGGCAGG (*BamHI*),

The PCR-amplified product was digested with the indicated restriction enzymes and inserted into the β-actin long promoter vector [12] thus generating a Dendra2 - β-actin (D2BA) fusion protein.

For generation of the β-actin – Dendra2 (BAD2) fusion protein, the β-actin cDNA was amplified using the following primers:

Forward: GAGCTC*AAGCTT*CGAATTCC (*HindIII*) and

Reverse: AAATTT*GGATCC*CGGAAGCATTTGC (*BamHI*), and inserted into the pDendra2-N vector using HindIII and BamHI restriction sites.

### Cells

RAW/LR5 macrophages [13] were maintained in RPMI 1640 supplemented with 10% newborn calf serum and 1% penicillin/streptomycin. For generation of a stably expressing population, cells were transfected with the Dendra2 - β-actin construct and selected against G418 (1 mg/ml). Stably expressing cells were further sorted by FACS to ensure homogenous expression of the construct. For experiments, RAW/LR5 cells were switched to phenol red – free RPMI 1640 medium supplemented with 10% newborn calf serum, L-glutamine and 25 mM HEPES pH7.4 [14].

MTLn3 rat mammary adenocarcinoma cells [15] were maintained in αMEM with 5% FBS and 0.5% penicillin-streptomycin mix. Cells were transfected with the Dendra2 - β-actin constructs 28 h prior to image acquisition using Lipofectamine 2000 (Invitrogen, Carlsbad, CA) and transferred to coated glass-bottomed dishes (Mattek) 22 h prior to image acquisition. Dishes were coated with 0.2% gelatin and cross-linked with 0.5% glutaraldehyde, resulting in a ∼100 nm thick matrix layer, which allows the monitoring of invadopodia dynamics without removing it from the evanescent field [16]. Cells were then serum-depleted in starvation media (L15, 0.8% BSA), for 3–4 hours prior to image acquisition.

### TIRF microscopy, data acquisition and image analysis

Acquisition of TIRF data was accomplished with a modified, commercial, objective-based TIRF Olympus IX71 microscope (Single Illuminator TIRFM, Olympus) (Supplementary Figure 1). In this system the original light sources were replaced by 5 lasers, three of which were used in our experiments: 407nm (CUBE, Coherent); 488nm (IMA Series, Melles Griot); 561nm (Jive 10, Cobolt). These free space lasers were coupled into a wide-band single mode fiber (kineFLEX, PointSource) connected to the TIRFM illuminator arm. Rapid switching and shuttering of the lasers was accomplished with an acousto-optic tunable filter (AOTFnC, AA Opto-electronic). Using custom designed mechanics, the TIRF angle adjustment micrometer was replaced by a high speed DC servo motor (M-235.2DD, Physik Instrumente) enabling the repeatable setting of different TIRF angles for each of the separate laser lines and rapid (<100 ms) switching between these TIRF positions and epifluorescent illumination for each line.

The objective used was a 150× 1.45NA TIRF (Olympus). Low light signal detection was accomplished using a deep-cooled, back-illuminated EMCCD camera (DU-897, Andor). The system was equipped with an environmental chamber kept at 37°C in a humidified atmosphere. Photoconversion was achieved by fully closing a field diaphragm within the illuminating arm, creating a circular exposed region (∼7 µm in diameter using the 150× objective), which can be positioned anywhere in the field of view, while keeping the rest of the field masked. A single 1 sec pulse of the 407 nm laser was administered at a region of the cytoplasm in the widefield mode. The diaphragm was then immediately opened and images acquired in TIRF mode at the rate of 0.5 frames/s for macrophages and 0.2 frames/s for carcinoma cells.

Images were collected using Metamorph software and image analysis was performed using ImageJ 1.43. Data were transferred to Microsoft Excel and MatLab for analysis of pixel intensity in macrophage podosomes or carcinoma cell invadopodial precursors. Only those podosomes that persisted through the imaging period and did not show significant lateral mobility nor underwent fusion or fission were analyzed. For carcinoma cells, analysis was performed on pre-existing invadopodial precursors as described previously [17]. Statistical significance was determined using Student's *t*-test. *P*≤0.05 was considered significant.

### Immunofluorescence microscopy

RAW macrophages or MTLn3 cells plated on thin collagen matrix were fixed in buffer containing 3.7% formaldehyde and permeabilized for 15 minutes in buffer with 0.15% saponin. Cells were stained with anti-β-actin antibody (AC-15; Sigma), followed by incubation with Alexa-488 conjugated anti-mouse secondary antibody and Alexa-647 conjugated phalloidin (Molecular Probes; Eugene OR). Images were acquired using the 60×/1.40NA objective of an Olympus IX71 microscope coupled to a Sensicam cooled CCD camera.

## Results

### Incorporation of photoconverted Dendra2-actin monomers in macrophage podosomes

**Figure 1:**
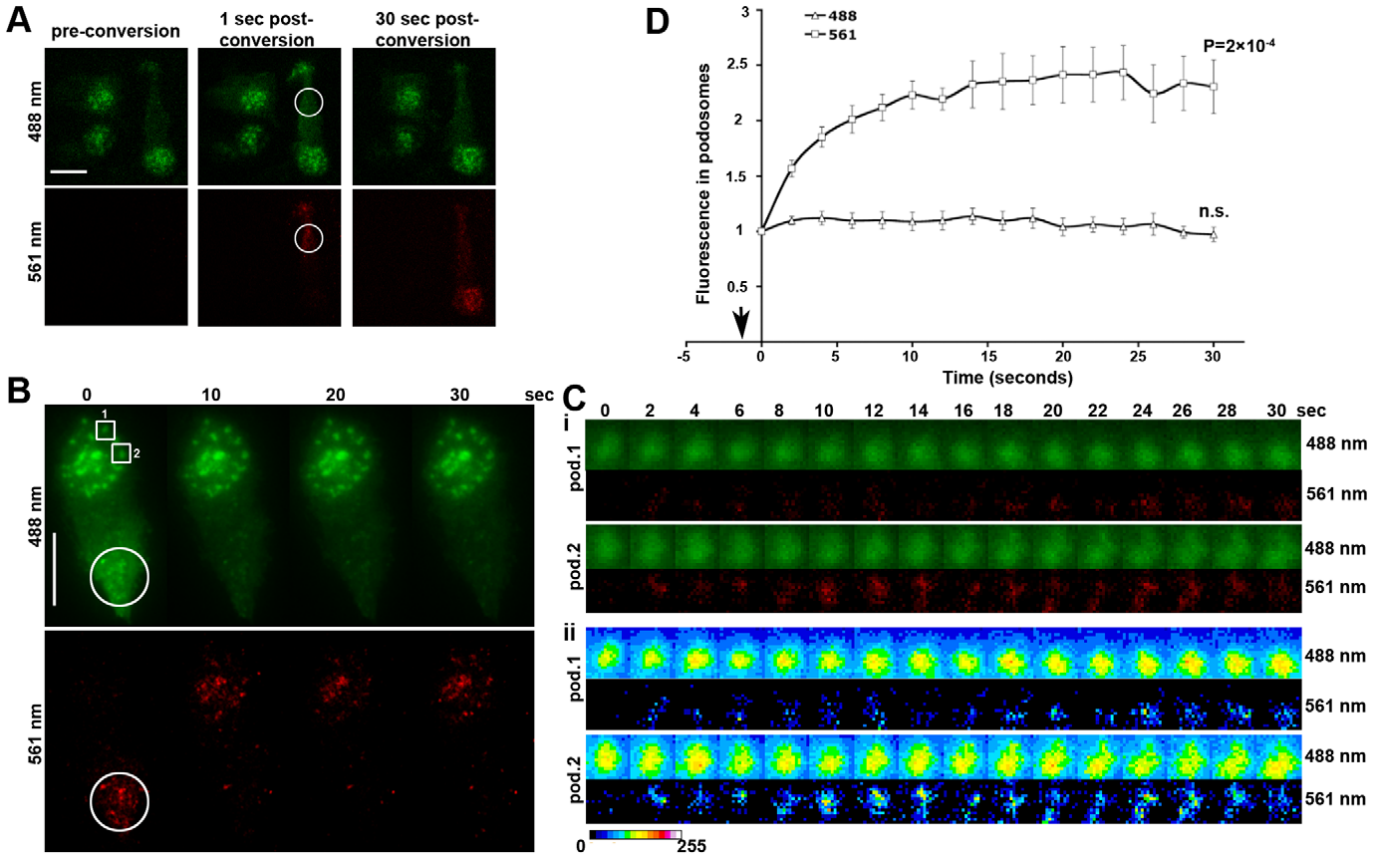
Incorporation of photoconverted Dendra2- β -actin monomers in macrophage podosomes under steady state conditions. (**A**) Photoconversion by utilizing a field diaphragm results in specific photoconversion of individual cells. TIRF images shown were acquired using 488 (green) and 561 (red) nm wavelengths before (pre), 1 sec and 30 sec after photoconversion. Conversion was achieved using a 407 nm laser on widefield mode for 1 second, resulting in conversion of the target cell at the encircled area, while leaving the neighboring cells intact. Scale bar, 10 µm. (**B**) Still TIRF images of a photoconverted cell at selected time points post-conversion (time 0 denotes the first frame acquired post-conversion), indicating the translocation of photoconverted D2BA species from the photoconverted area (circled) to podosomes (561 nm). Scale bar, 10 µm. (**C**) **i**. Blow-up of the podosomes indicated on panel B (boxes 1 & 2) during the entire post-conversion time-lapse showing rapid incorporation of photoconverted D2BA monomers into macrophage podosomes. **ii**. Pseudocolored pixel intensity images of the same podosomes. Pseudocolor scale - blue indicates low and red high intensity. Intensity of the photoconverted species increases inside podosomes indicating incorporation into filaments, while intensity of the non-converted species remains unaltered. (**D**) Kinetics of photoconverted D2BA incorporation into podosomes (n = 100 podosomes from 10 cells analyzed in two independent experiments). Pixel intensity analysis of podosomes reveals that the red species increase inside podosomes, while the green fluorescence intensity remains unaltered. The arrow indicates the time of the photoconversion (−1 second). No statistically significant changes in the green fluorescence in podosomes were observed at any time-point relative to time 0, unlike the red fluorescence where all points were significantly different to time 0 (shown is the *P* value for the 30 sec time-point).

Previous studies have successfully utilized GFP fused to the N-terminus of β-actin to monitor actin dynamics in living cells [9], [14], [17]. Therefore, we initially created an N-terminus Dendra2 fusion construct (D2BA). This construct was expressed in the RAW 264.7 macrophage cell line (RAW/LR5) and D2BA correctly localized to F-actin rich structures such as ruffles and podosomes (Figure 1A).

To test whether photoconverted D2BA would incorporate into macrophage podosomes under steady-state conditions, TIRF microscopy was employed in order to minimize the contribution of the cell body and eliminate out-of-focus light. We have previously demonstrated that active WASp, a major actin polymerization activator involved in podosome formation and dynamics, is visible by TIRF in macrophage podosomes [14], therefore, we reasoned actin polymerization should also be visible by TIRF. Mature leukocyte podosomes are dome-shaped [18] ventral adhesion structures and are thought to be in steady state through a continuous process of filament severing and actin polymerization [19], until they eventually disassemble. They therefore represent a model filamentous structure wherein the net polymerization and depolymerization rates are roughly equal, resulting in retention of the shape and F-actin content of the podosome at steady state.

RAW macrophages stably expressing D2BA under the human β-actin promoter were grown on glass-bottomed dishes and imaged by TIRF. Photo-conversion of D2BA monomers was performed in a region of the cell devoid of podosomes and was achieved by switching from TIRF to widefield, while use of a field diaphragm allowed 407 nm laser light to illuminate a region of 7 µm in diameter in the field of view creating a cytoplasmic pool of actin monomers through the cell body within the selected region. This resulted in rapid and highly selective photoconversion, leaving neighboring cells unaffected (Figure 1A). Subsequently, incorporation of the photoconverted D2BA monomers into podosomes was imaged in TIRF. Photoconverted G-actin was incorporated into podosomes from regions that extended more than 15 µm behind the podosome region and was evident 2 seconds after a 1-second photoconversion pulse, suggesting rapid delivery of the converted actin monomers at speeds in excess of 5 µm/sec (Figure 1B and 1C). This concurs with the values reported by Zicha et al [8]. Incorporation of photoconverted actin into podosomes was rapid and reached a signal plateau by ∼10 seconds (Figure 1D), which is in agreement with actin recovery rates obtained by FRAP of macrophage podosomes expressing EGFP-β-actin [14]. This also indicated that the actin monomers that contribute to podosome filaments originate from distant parts in the cell body and are not derived from local pools. By contrast, in the green channel fluorescence intensity remained unaltered, suggestive of steady-state podosomes. Furthermore, only a fraction of the green D2BA was photoconverted in the selected region, therefore the red D2BA could not substitute the green D2BA in podosomes. In conclusion, the increase in red incorporation does not depend on a burst of actin polymerization but occurs at steady state and is only visible through the use of the photoconverted Dendra2-β-actin fusion protein.

### Incorporation of photoconverted D2BA monomers occurs at barbed ends

**Figure 2:**
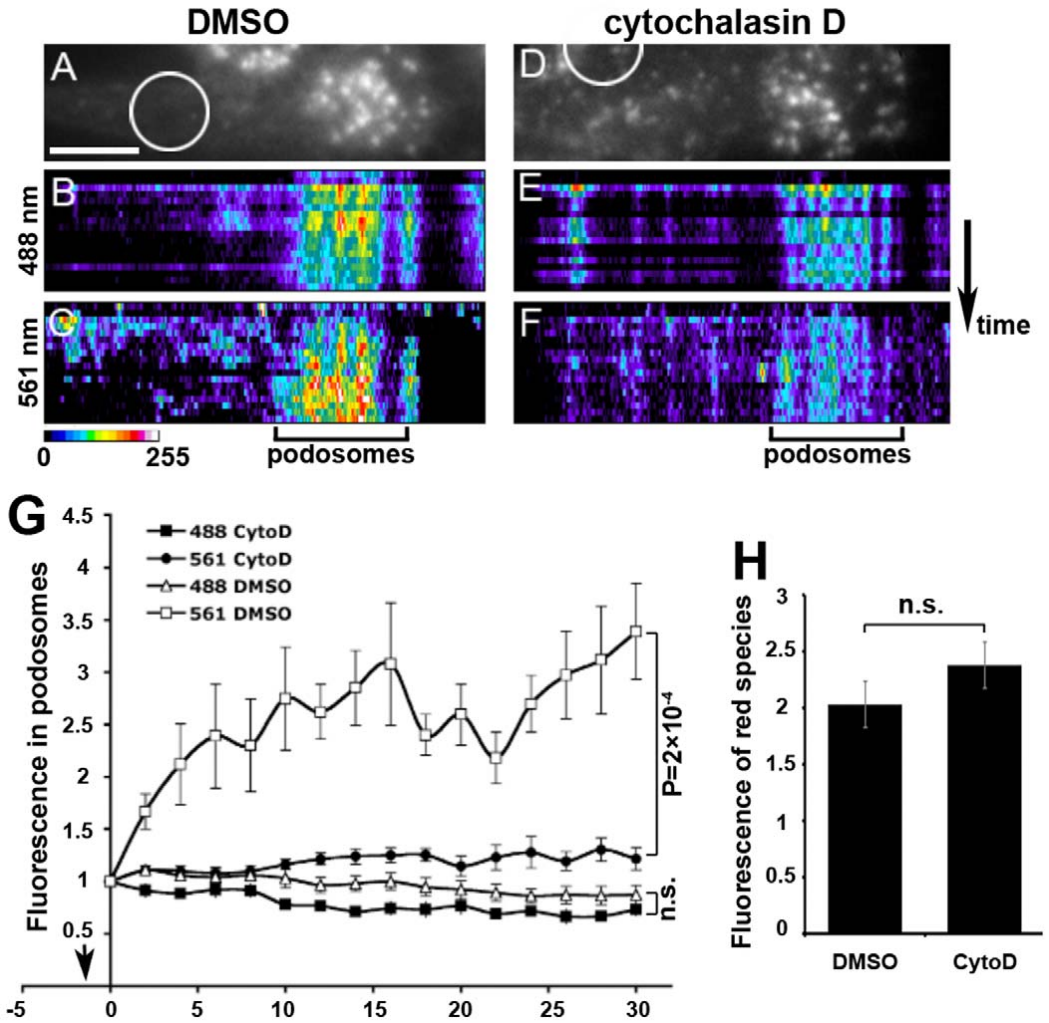
Inhibition of incorporation of photoconverted D2BA into macrophage podosomes by cytochalasin D. (**A–F**) Cells were treated with DMSO (A–C) or 2µM cytochalasin D (D–F) and D2BA was photoconverted in the indicated areas (circled). Kymographs of pseudocolor pixel intensity indicate that there is no incorporation of photoconverted D2BA in the podosomes of cytochalasin D compared to DMSO-treated cells. Scale bar, 10 µm. Pseudocolor scale - blue indicates low and red high intensity. (**G**) Graph indicating the kinetics of D2BA incorporation into podosomes of DMSO or cytochalasin D-treated cells. n = 80 podosomes from 8 cells per condition analysed in two independent experiments. Cytochalasin D treatment effectively blocks incorporation of D2BA into podosomes, indicating that polymerization occurs at barbed ends in podosomes. Statistical analysis was performed at the 30 sec time point comparing intensities in podosomes between cells that received DMSO and those that received cytochalasin D; n.s., not significant. (**H**) The amount of the total photoconverted species (561 nm) is not altered between DMSO or cytochalasin D treated cells. Pixel intensity at 561 nm was analyzed in widefield in order to capture the photoconverted species of the entire cell; n = 7 cells from a representative experiment.

In order to investigate if D2BA incorporation occurred specifically at barbed ends, cells were pretreated with cytochalasin D, a barbed end blocker, and incorporation of photoconverted D2BA monomers into podosomes was imaged. Macrophages were incubated with cytochalasin D for 4 to 6 minutes, which was found to be a critical time range. At this time, podosomes were present and could be imaged by TIRF, but incorporation of actin was eliminated or significantly reduced (Figure 2A–G). Incubation times shorter than 4 minutes resulted in actin incorporation, while after incubation of more than 7 minutes most cells had disassembled their podosomes (data not shown and [14]). As shown in Figure 2A, in the presence of cytochalasin D, photoconverted D2BA was not incorporated in podosomes, indicating that cytochalasin D effectively blocked barbed ends at podosomes. The green signal in pre-existing podosomes was unaltered indicating that podosomes were neither growing nor disassembling. The modest increase in the intensity of the red signal in podosome areas was not due to incorporation of photoconverted actin into podosomes but, more likely, to diffusion of photoconverted monomers into the podosome area. Importantly, treatment of cells with cytochalasin D did not affect the amount of D2BA being photoconverted (Figure 2H), while the rate of photoconverted D2BA leaving the region of photoconversion was roughly equal between DMSO- and cytochalasin D – treated cells (not shown). Cytochalasin D, therefore did not affect D2BA dynamics in the time-range selected and effectively blocked its incorporation in podosome barbed ends.

### The orientation of Dendra2 relative to β-actin affects the distribution of the Dendra-actin fusion protein

**Figure 3:**
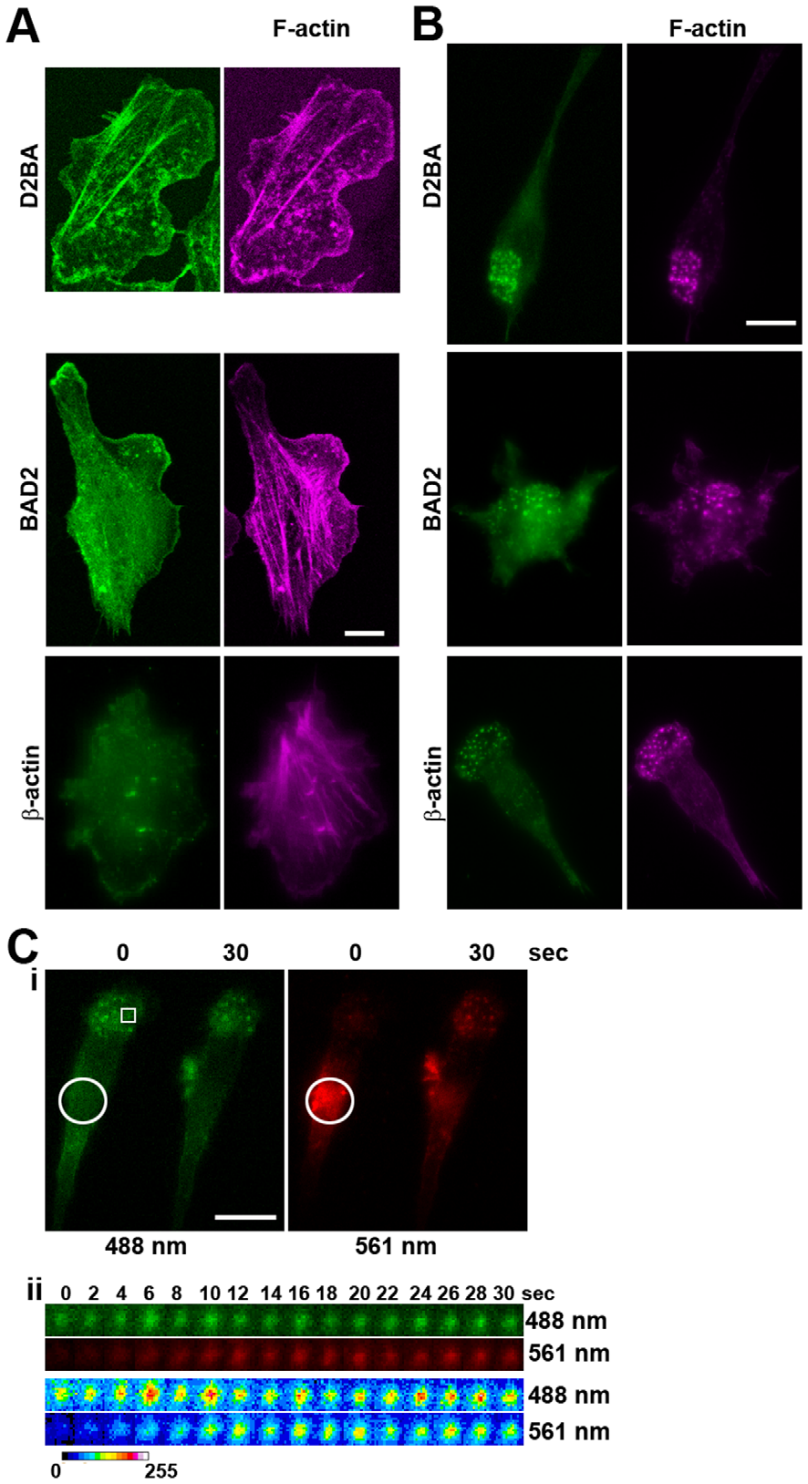
The orientation of the Dendra2 fluorescent protein relative to β-actin affects the localization of the fusion protein in carcinoma, but not macrophage, cells. (**A, B**) MTLn3 cells (**A**) or RAW macrophages (**B**) were transfected with either D2BA (upper panels), BAD2 (middle panels) or fixed and stained with an antibody against endogenous β-actin (lower panels) and stained for F-actin using phalloidin-Alexa 647. In MTLn3 cells, the D2BA construct localizes to stress fibers, lamellipodia and invadopodia, whereas the BAD2 construct localizes to cytoplasm, lamellipodia and invadopodia, similar to endogenous β-actin, without affecting endogenous stress fibers. In macrophages, both constructs show indistinguishable localization with endogenous β-actin and F-actin. (**C**) Incorporation of photoconverted BAD2 in macrophage podosomes is similar to that observed with D2BA. **i**. Still images of a cell expressing BAD2 photoconverted in the encircled region in widefield at 0 and 30 seconds after photoconversion. **ii**. Blow-up of the podosome indicated in **i** during the entire post-conversion time-lapse showing rapid incorporation of photoconverted BAD2 monomers into the podosome. Scale bars, 5 µm (A) and10 µm (B, C).

In order to observe actin polymerization in invadopodia using the Dendra2-β-actin method we employed the same Dendra2-β-actin N-terminal fusion protein, D2BA, used in macrophages above. However, fluorescence from invadopodial precursors was complicated by incorporation of D2BA into stress fibers, which partially obscured the invadopodial precursors (Figure 3A). Since stress fibers are preferentially composed of γ-actin while branched dynamic filament populations found in protrusions are composed of β-actin (reviewed in [20]) this indicated that N-terminal tagging of β-actin alters its localization. In this regard, it has recently been shown that the localization of β-actin is dependent on N-terminal post-translational modifications, such as arginylation [21] and suggests that post-translational modifications may be prevented when a fluorescent protein is fused to its N-terminus. Therefore, we prepared a β-actin-Dendra2 C-terminal fusion construct (BAD2) that would allow correct N-terminal post-translational modifications to take place. D2BA expressed in carcinoma cells was incorporated in stress fibers like that reported for endogenous γ-actin [22] (Figure 3A, upper panel). However, BAD2, like endogenous β-actin, was found in areas of protrusion, [21], and in invadopodia but not in stress fibers, which were still present (Figure 3A, middle and lower panels). Additionally, BAD2 was correctly localized to EGF-induced lamellipodia (Supplementary Figure 2). Similar localization patterns were observed when EGFP was fused to the N- or the C-terminus of β-actin indicating that these results are not specific to Dendra2 fusions (X.C. and J.C., data not shown). Localization of BAD2 and D2BA in macrophages was indistinguishable, most likely due to the absence of stress fibers in these cells (Figure 3B) and matched that of endogenous β-actin (Figure 3B). Additionally, photoconverted BAD2 showed similar kinetics of incorporation into podosomes with D2BA (Figure 3C), indicating that the orientation of the fluorescent protein does not affect the behavior of the fusion protein in macrophages. These results suggest that the β-actin-Dendra2 C-terminal fusion protein, BAD2, is correctly localized similar to that of endogenous β-actin, in carcinoma cells. Therefore, we used the BAD2 construct for further studies of actin polymerization in invadopodia.

### Incorporation of photoconverted β-actin-Dendra2 monomers into invadopodium precursors of carcinoma cells

**Figure 4:**
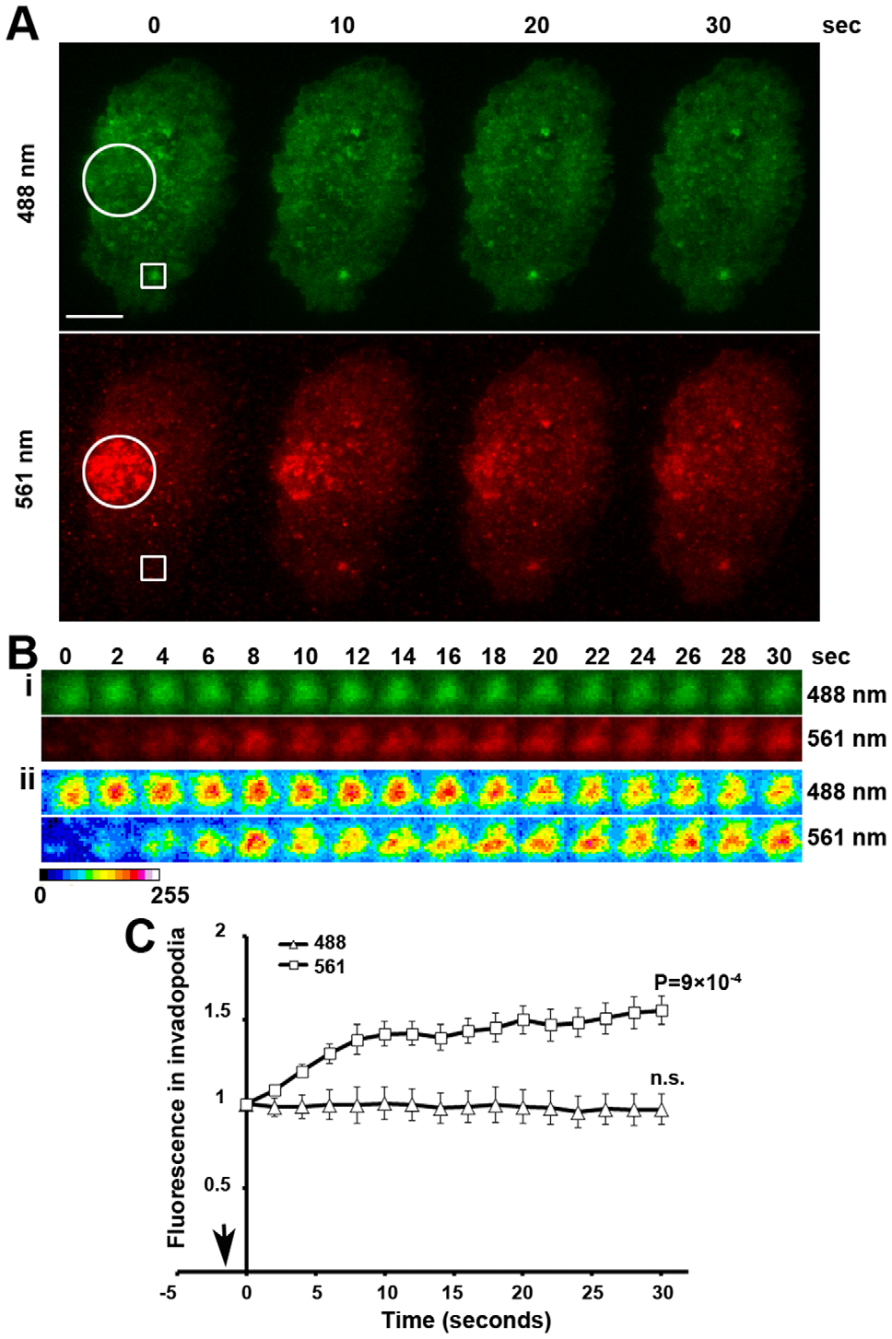
Constitutive incorporation of photoconverted β-actin-Dendra2 monomers in MTLn3 cancer cells under steady state conditions. (**A**) Still images of a photoconverted MTLn3 cell at selected time points post-conversion (time 0 denotes the first frame acquired post-conversion), indicating the translocation of photoconverted D2BA species from the photoconverted area (circled) into an invadopodium (boxed region) in 561 nm. Scale bar, 5 µm. (**B**) **i**. Blow-up of the invadopodium boxed in panel A showing rapid incorporation of photoconverted BAD2 monomers into invadopodial F-actin. **ii**. Pseudocoloured pixel intensity images of the same invadopodium. Intensity of the photoconverted species increases inside the invadopodium indicating incorporation into filaments, while intensity of the non-converted species remains constant. (**C**) Kinetics of photoconverted actin incorporation into invadopodia. Intensity of the red species in invadopodia increases under steady-state conditions while the intensity of the green species remains unaltered. N = 11 invadopodia from 8 different cells. No statistically significant changes in the green fluorescence in invadopodial precursors were observed at any time-point relative to time 0, unlike the red fluorescence where all points were significantly different to time 0 (shown is the *P* value for the 30 sec time-point).

Under steady state conditions MTLn3 rat mammary adenocarcinoma cells plated on a thin gelatin substrate exhibit continuous incorporation of BAD2 into invadopodial precursors at rates similar to those of macrophage podosomes (Figure 4; compare with Figure 1D). Green fluorescence remains stable, as actin polymerization and depolymerization go on at similar rates (Figure 4). In order to shift the relative balance of the filaments in invadopodial precursors towards increased actin polymerization we treated serum-deprived MTLn3 cells with EGF. We have previously shown using the time-resolved fixation method that EGF stimulation increased the barbed ends of invadopodial precursors [17]. This increase in barbed ends reached a peak at 1 minute post-stimulation, with free barbed ends persisting up to 3 minutes, at which time they returned to basal levels [17]. The EGFP- β-actin method was able to capture the initial 1-minute peak in live cells, however it was not able to demonstrate continuing actin polymerization past that point [17] as the presence of free barbed ends would predict.

**Figure 5:**
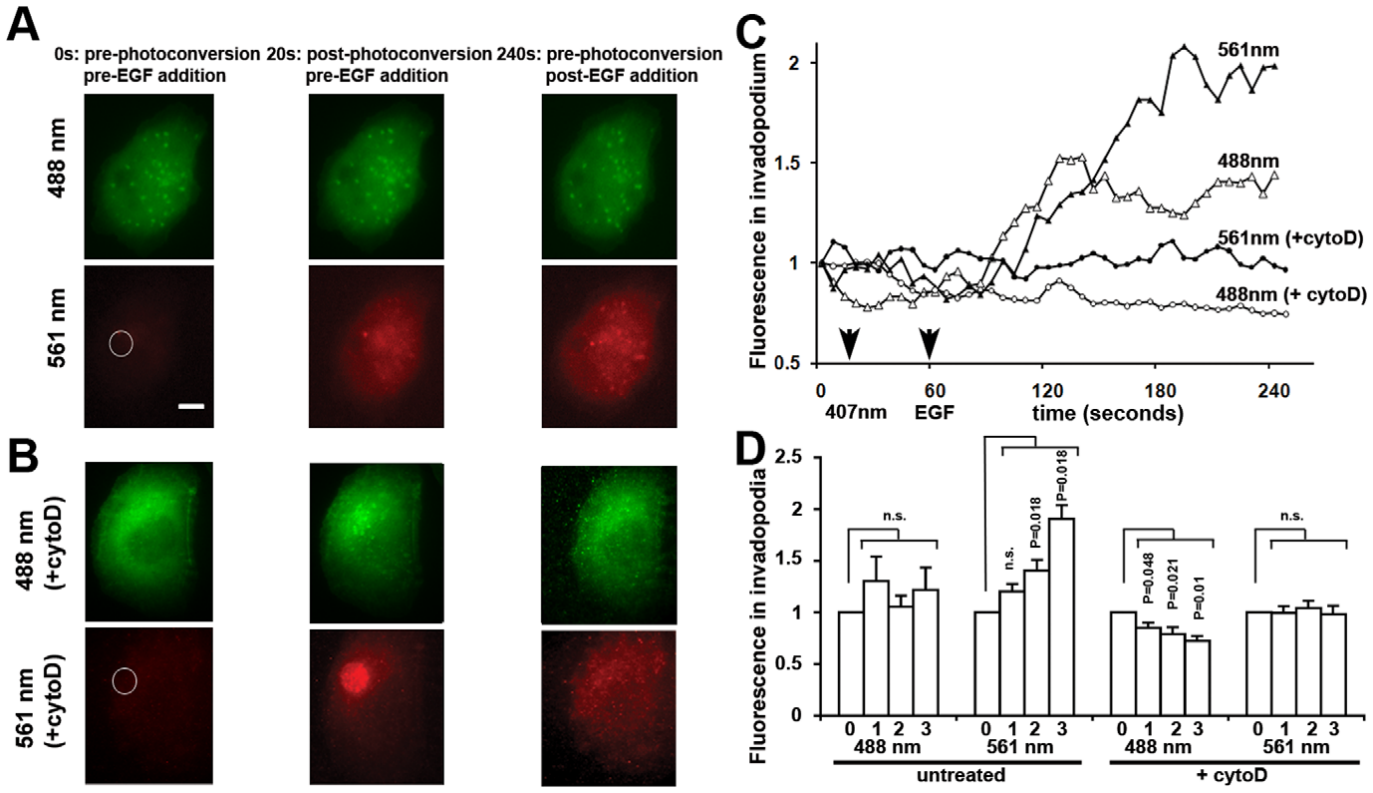
Photoconversion of β-actin-Dendra2 monomers in MTLn3 cells reveals extended actin polymerization at barbed ends in invadopodial precursors stimulated by EGF. (**A,B**) Still images of cells that were either left untreated (A) or treated with cytochalasin D (B) taken before the photoconversion (0s), after photoconversion (20s) and after EGF stimulation (240s). Each cell is shown in green, 488nm channel (upper panels) and red, 561nm channel (lower panels). White circle marks the site of photoconversion. Scale bar, 2µm. (**C**) Representative plots of actin fluorescence of unconverted (488 nm) and photoconverted (561 nm) BAD2 species in an invadopodium of an untreated and a cytochalasin D (cytoD)-treated cell. Arrows point to the time of photoconversion (407 nm) and EGF addition. Cytochalasin D blocks EGF-induced BAD2 incorporation into the invadopodium. (**D**) Average fluorescence intensity of unconverted (488 nm) and photoconverted (561 nm) BAD2 in invadopodia of cells that were left untreated or treated with cytochalasin D (+cytoD). 0: pre-EGF addition; 1: 1 min after EGF addition; 2: 2 min after EGF addition; 3: 3 min after EGF addition. Values were normalized to pre-photoconversion values and average of intensity in invadopodial precursors from 5 different cells per condition are shown +/−s.e.m.

We repeated this experiment in MTLn3 cells transfected with BAD2 plated on thin (100 nm) gelatin matrix [16] and serum-starved prior to imaging. A cytoplasmic region with no visible invadopodial precursors was photoconverted and 20-25 seconds later, red BAD2 monomers were homogeneously distributed in the cytoplasm but did not incorporate efficiently into invadopodial precursors (Figure 5A), suggesting that actin filaments in invadopodial precursors of quiescent serum-deprived cells may be capped. Subsequent EGF stimulation resulted in an increase of the intensity in both green (non-converted) and red (photoconverted) BAD2 species at pre-existing invadopodial precursors. However, whereas the green species reached a plateau of intensity at 1 minute post-stimulation, the red species continued to increase, plateauing at 3 minutes (Figure 5 C, D). This indicates that incorporation of actin monomers onto free barbed ends continues up to 3 minutes post-EGF stimulation. Similar to macrophage podosomes, incorporation of photoconverted BAD2 monomers was inhibited in cells that had been pre-treated with cytochalasin D for 1 minute before the photoconversion and EGF stimulation (Figure 5B,C, D) suggesting that incorporation of BAD2 occurred in barbed ends of growing invadopodial precursors. We have been able therefore to measure extended actin incorporation into free barbed ends without the complexity of depolymerization.

## Discussion

In this study, we have developed and presented a method to directly visualize cytochalasin D-sensitive actin incorporation into the free barbed ends of pre-existing filamentous actin structures, demonstrated in macrophage podosomes and carcinoma cell invadopodial precursors. This method combines the photoconversion of a subpopulation of Dendra2-β-actin monomers in the cytoplasm in widefield, followed by imaging of molecular movements using TIRF microscopy, which increases the signal-to-noise ratio in ventral, invasive F-actin-rich structures because it restricts image acquisition to a single focal plane and is unaffected by the relative volume of the fluorescently labeled actin within the cytoplasm above the podosome or invadopodium. Using this technique we have demonstrated β-actin incorporation into free podosome barbed-ends at steady state, without stimulation of actin polymerization, an effect that is not observed when a single-wavelength fluorescent protein is used unless photobleaching techniques are employed. Advantages of photoconversion over FRAP however are the elimination of photo-damage artifacts induced by high laser power photobleaching and the bleaching of pools of G actin that may contribute to the recovery of the bleached area, while a prior assumption as to the location of barbed ends is not required.

Another major advantage of this technique over previously described approaches is that it is performed in live cells without the need for potentially deleterious permeabilization steps, and it extends measurements of actin growth in barbed ends well beyond initial transients, because it circumvents fluctuations in fluorescence resulting from depolymerization of the pre-existing filament, which is still labeled with the green, non-photoconverted species. This is evident in our measurements of actin filament growth in carcinoma cell invadopodial precursors. While we have previously shown using a fixed cell method that barbed ends in invadopodium precursors persist for 3 minutes following EGF stimulation, only the initial peak of barbed end formation at 1 minute post-EGF stimulation could be measured using GFP-β-actin intensity in live cells [17]. Use of the BAD2 construct enabled us to visualize β-actin incorporation past that initial peak consistent with persistence of free barbed ends in invadopodium precursors up to 3 minutes.

One important consideration however, is selection of the area in which photoconversion will occur. In this study we chose to convert the highly mobile monomeric G-actin in both macrophages and carcinoma cells in order to image its incorporation in areas of active polymerization. For this reason, we had to take into account the orientation of Dendra2 relative to β-actin. While in macrophages β-actin-Dendra2 fusion proteins showed similar distribution in the cell body irrespective of whether Dendra2 was fused to the N- or C-terminus of the β-actin cDNA, in carcinoma cells Dendra2 had to be fused to the C-terminus of β-actin so as to prevent its sequestration from the cytoplasm and its incorporation into stable stress fibers, which are not present in macrophages. Indeed, the use of D2BA in carcinoma cells was problematic for the following reasons: prominent stress fibers obscure the visualizing of invadopodia; and actin in stress fibers is largely immobile [23], therefore photoconversion of MTLn3 cells expressing D2BA would yield photocoverted actin that would not be freely diffusible to incorporate efficiently into other F-actin structures. We hypothesise that the differential localization of the D2BA and BAD2 constructs is due to the lack of N-terminal post-translational modifications in the D2BA construct such as arginylation, which affects the bundling properties of β-actin and promotes its preferential incorporation into branched - rather than bundled - filaments [21], [24], and potentially other non-filamentous actin-containing particles [25]. Therefore, the localization of the BAD2 construct more closely resembles that of endogenous β-actin (Figure 3).

The use of Dendra2-β-actin constructs can be extended to any of the cell compartments with active actin dynamics, such as leading edge or filopodia. Simultaneous measurements of actin incorporation rates in different compartments can be done within the same cell. In addition, application of D2BA and BAD2 need not be limited to study areas of active polymerization but may also be used to measure diffusion from a specific point and identify the inter-relationship of β-actin molecules between different sub-cellular compartments. An example would be testing if a gradient of chemoattractant induces mobilization of photoconverted β-actin monomers from one compartment to a different one.

## Supporting Information

Figure S1 **Layout of the TIRF microscope.** The light from five free-space lasers are combined using dichroic mirrors, sent through an AOTF and coupled into a single mode fiber optic cable. The output end of the fiber is mounted on a translation stage controlled by a high speed DC servo motor. The aperture of the fiber is imaged to the back focal plane of an objective lens using a telescope containing an adjustable field diaphragm. Generated fluorescence is collected by the same objective lens and imaged onto a deep-cooled EMCCD camera.
(TIF)

Figure S2 **Incorporation of photoconverted BAD2 in lamellipodia of EGF-stimulated MTLn3 cells.** BAD2 expressed in MTLn3 cells were photo-converted by a 0.7-second pulse of 407 nm illumination on a DeltaVision imaging system prior to EGF addition (time 0), resulting in photoconversion of BAD2 in the entire cell volume. EGF was subsequently added and cells were monitored over time on widefield mode. Shown are still images of the photoconverted cells prior to EGF addition (time 0) and 1 minute after EGF stimulation. Photoconverted BAD2 is incorporated in the barbed ends of the protruding lamellipodium (arrowheads; 1 min.). Scale bar, 5 µm.
(TIF)
