# Supplementary figures and images for: Visualization of Actin Polymerization in Invasive Structures of Macrophages and Carcinoma Cells Using Photoconvertible β-Actin – Dendra2 Fusion Proteins
Source: PLoS One. 2011 Feb 14;6(2):e16485. doi: 10.1371/journal.pone.0016485 (PMC3038862; doi:10.1371/journal.pone.0016485)

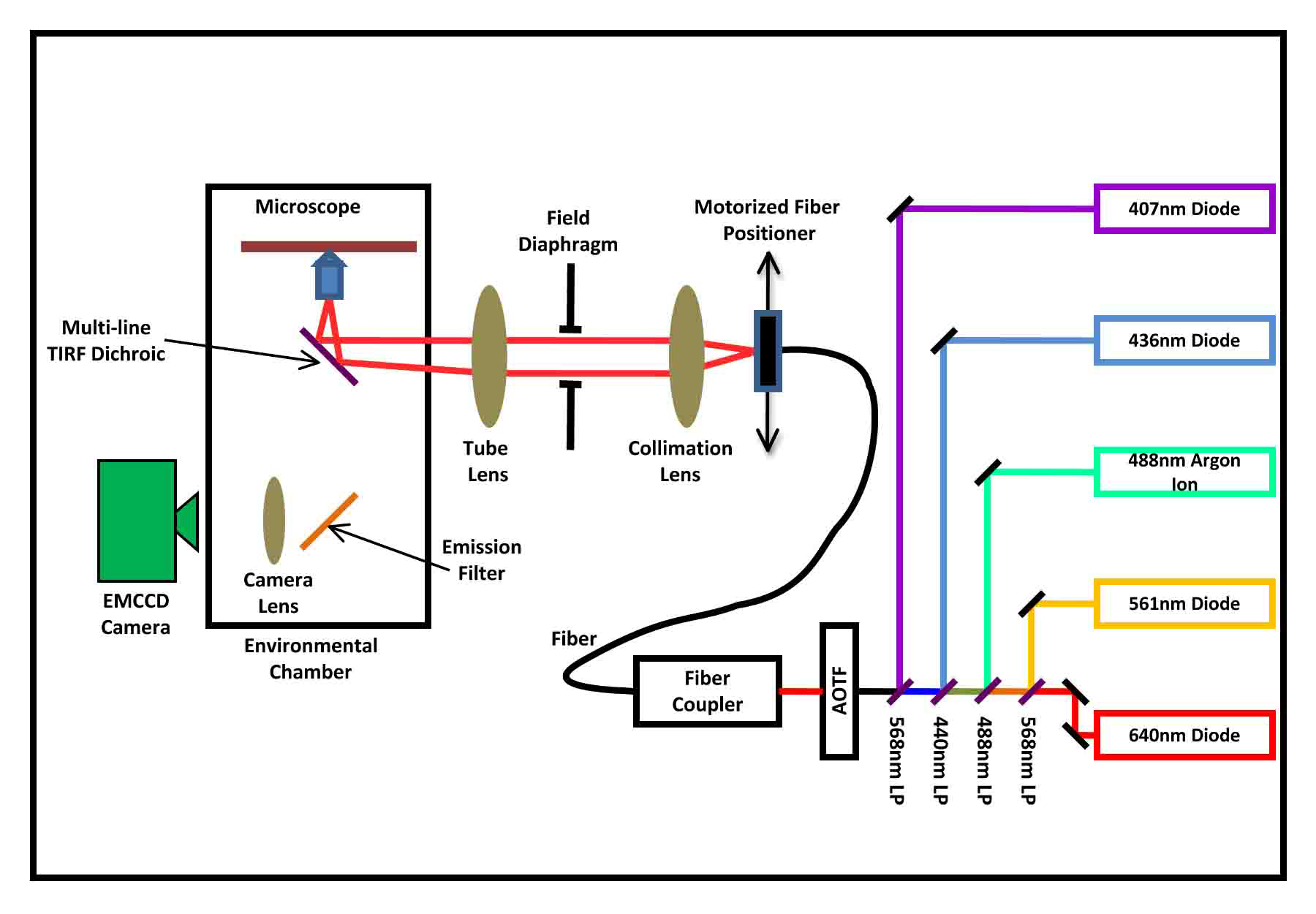

Supplement: Figure S1 — Layout of the TIRF microscope. The light from five free-space lasers are combined using dichroic mirrors, sent through an AOTF and coupled into a single mode fiber optic cable. The output end of the fiber is mounted on a translation stage controlled by a high speed DC servo motor. The aperture of the fiber is imaged to the back focal plane of an objective lens using a telescope containing an adjustable field diaphragm. Generated fluorescence is collected by the same objective lens and imaged onto a deep-cooled EMCCD camera. (TIF) [file pone.0016485.s001.tif]

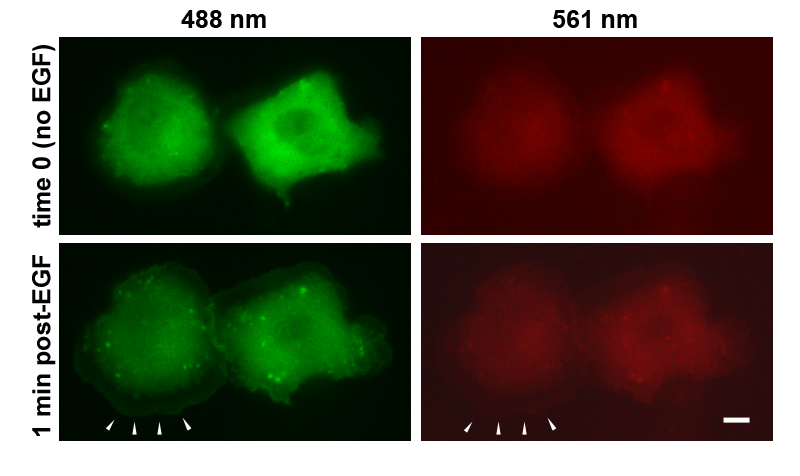

Supplement: Figure S2 — Incorporation of photoconverted BAD2 in lamellipodia of EGF-stimulated MTLn3 cells. BAD2 expressed in MTLn3 cells were photo-converted by a 0.7-second pulse of 407 nm illumination on a DeltaVision imaging system prior to EGF addition (time 0), resulting in photoconversion of BAD2 in the entire cell volume. EGF was subsequently added and cells were monitored over time on widefield mode. Shown are still images of the photoconverted cells prior to EGF addition (time 0) and 1 minute after EGF stimulation. Photoconverted BAD2 is incorporated in the barbed ends of the protruding lamellipodium (arrowheads; 1 min.). Scale bar, 5 µm. (TIF) [file pone.0016485.s002.tif]
